# The role of death-associated protein 3 in apoptosis, anoikis and human cancer

**DOI:** 10.1186/s12935-015-0187-z

**Published:** 2015-04-10

**Authors:** Umar Wazir, Mona MAW Orakzai, Zubair S Khanzada, Wen G Jiang, Anup K Sharma, Abdul Kasem, Kefah Mokbel

**Affiliations:** The London Breast Institute, Princess Grace Hospital, London, UK; Department of Breast Surgery, St. George’s Hospital and Medical School, University of London, London, UK; Ayub Medical College, Abbottabad, Pakistan; Metastasis and Angiogenesis Research Group, University Department of Surgery, Cardiff University School of Medicine, Cardiff University, Cardiff, Wales UK

**Keywords:** Apoptosis, Review, Death-associated protein 3, Anoikis, Cancer, Carcinogenesis, Oncogenesis, Breast cancer

## Abstract

Death-associated protein 3 (DAP3) is a molecule with a significant role in the control of both apoptosis and anoikis. Apoptosis is the predominant type of programmed cell death (PCD) which may occur in response to irreparable damage to DNA, or in response to induction by inflammatory cells. Anoikis is subset of apoptosis which occurs in epithelial cells in response to detachment from the surrounding matrix. Both apoptosis and anoikis are of interest in the context of carcinogenesis. In this review, we shall discuss apoptosis and anoikis, and the recent literature regarding the role of DAP3 in both these pathways.

## Background

Apoptosis is one of the several mechanisms of programmed cell death (PCD) which may occur in response to irreparable damage to DNA, or when induced by inflammatory cells. It is an active, ATP dependent process, as opposed to necrosis, which is a passive process of cell destruction. It is distinct from necrosis, which may also be referred to as accidental cell death (ACD). Histologically, necrosis is characterised by karyolysis and cell swelling, whilst the stigmata of apoptosis include shrinkage of the nucleus (pyknosis) and cytosol, with a virtual absence of inflammation [1,2].

The existence of programmed cell death was initially proposed by Gluckman (1951) [3]. Further investigation into cell death discerned at least two distinct patterns. In addition to the better known necrosis, histological studies in hepatic ischaemia were suggestive of a so-called ‘shrinking necrosis’, which was distinct from the more typical necrosis [4]. Kerr *et al.* recognised this to be distinct from canonical necrosis, coined the term ‘apoptosis’ for this process, and noted its role in embryogenesis, teratogenesis, tumour response, and normal tissue turnover in response to hormonal factors [5].

It is suggested that apoptosis and necrosis may be two extremes of a continuum of cell demise. Deleterious stimuli of insufficient magnitude to cause necrosis may cause involution of tissue by way of apoptosis. As noted above, this was the original context in which apoptosis was identified [6,7]. The essential distinction between the two processes has been stated to be that, whilst necrosis is typically a passive process, apoptosis is an energy-dependent phenomenon requiring an expenditure of ATP [8].

The processes underlying apoptosis have been implicated in carcinogenesis and have thus been the subject of continued research in the context of cancer. The earliest and most well − documented apoptosis gene is the so-called ‘guardian of the genome’, tumour protein 53 (p53*).* It was initially identified in animal models in 1979 in association with SV40 large T antigen, and was dubbed p53 reflecting its molecular weight of 53 kDa [9,10]. Subsequent studies found it to be over expressed in cells transformed by chemical carcinogens or ultra-violet radiation [11-13]. Wild type p53 was found to be a tumour suppressor, with a role in cell cycle progression, apoptosis, and the cell response to DNA damage. Mutation of p53 have been implicated in neoplastic transformation in a number of models [11].

Since then, a number of other genes have been studied for their role in programmed cell death including, but not limited to, B-cell lymphoma 2 protein (BCL2*),* the BCL2 associated X protein (BAX*)* [14] and those encoding caspases 3, 6, 8 and 9 [15].

Anoikis is a relatively novel area of research in the field of PCD that has also been of great interest in the context of carcinogenesis. Specifically, it is the invocation of apoptosis in response to cell detachment from the extra cellular matrix (ECM), especially in the context of parenchymal cells [16].

The ability to survive detachment from the ECM is prerequisite for the transition to oncogenesis. This makes anoikis a significant area of interest for cancer research [17].

Our understanding of the apoptosis pathway continues to evolve. Two major canonical pathways have been identified, which terminate in a common execution pathway. In addition, a further pathway bypassing the common execution cascade is also discussed below. Finally, we shall also review the literature regarding anoikis and the specific programmes within apoptosis that contribute to it.

### Extrinsic pathway

This pathway is mediated by the activation of trans-membranous receptors and is commonly induced by inflammatory cells in response to viral infection or oncogenesis. These receptors are part of the tumour necrosis factor receptor (TNFR) super family, six of whom have been characterised as death receptors (DR). These receptors have been characterised as type I transmembrane protein, with an N-terminal cytoplasmic terminus. This end of the protein contains an active motif termed a ‘death domain’ (DD), which interacts with adapter proteins, thus initiating intra-cellular pathways. *In vivo*, the ligands for these receptors have been identified as TNF-α, the Fas ligand (Fas-L), TNF related apoptosis inducing ligand (TRAIL), as well as other members of the TNF family. The best documented of these receptors are TNFR1 and Fas (DR1 and DR2). Others include TRAIL-R1 and 2 (DR 4 and DR5), and DR3 and DR6 [18].

### Death Inducing Signalling Complex (DISC)

The intra-cellular signalling complex for DRs has been designated as the death inducing signalling complex (DISC). Broadly speaking, the DR mediates its actions when activated by interaction with its respective ligand. The DR typically undergoes trimerisation when thus activated. The DD of the receptor interacts with the DD on the FAS Associated Death Domain (FADD) protein, which is normally localised in the nucleus. In turn, the FADD interacts with pro-caspase 8, which is also known as FADD-like interleukin-1 beta-converting enzyme (FLICE). These interactions are mediated by corresponding death effector domains (DED) on FADD and FLICE, leading to the activation of the apical caspase 8 [19]. This interaction is inhibited by FLICE-like inhibitory protein (FLIP), which competes with FLICE by interacting with FADD [20].

The DISCs of the aforementioned six DRs have small but significant variations between themselves whilst retaining the general plan described above. It would be reasonable to surmise that these variations enable variations in the downstream effects of each of the six DRs.

The most significant variation can be seen in the context of FAS and the two TRAIL receptors. In addition to pro-caspase 8, they also activate pro-caspase 10. The downstream effects of caspase 10 are believed to be largely similar to those of caspase 8, thus providing a redundant signalling pathway [21,22].

Furthermore, the TNFR DISC requires a DD-bearing adaptor protein in addition to FADD, referred to as TNFRSF1A-associated via death domain (TRADD). TRADD is required for FADD-mediated apoptosis. In addition, TRADD also mediates interactions with TNF receptor-associated factor 2 (TRAF2), which mediates immune effects of TNF. Specifically, it activates the nuclear factor of kappa light polypeptide gene enhancer in B-cells 1 (NFKB1), which is involved in the control of genes responsible for inflammation and immune response [23].

### DAP3 and DRs

**Figure 1:**
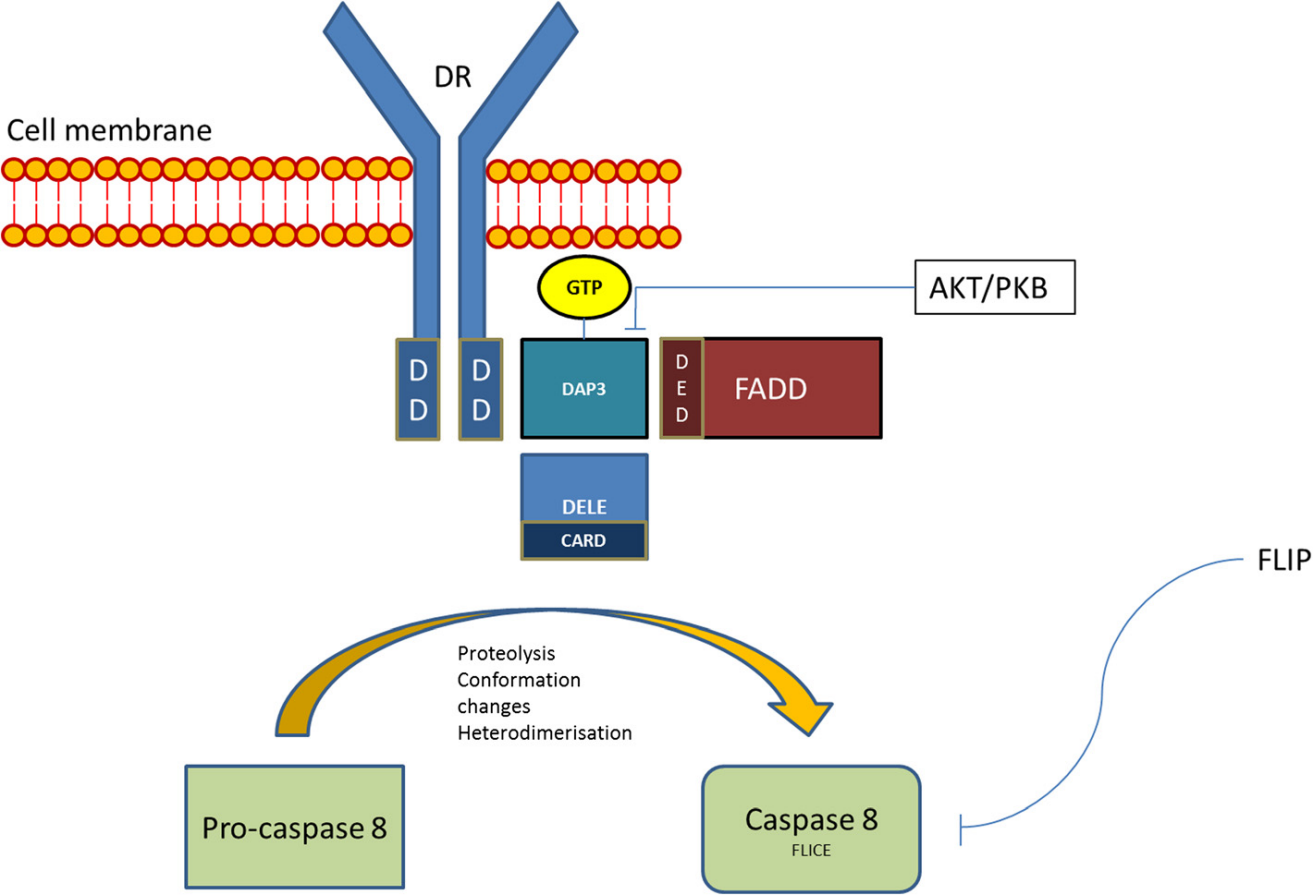
**Schematic representation of the relation of DAP3 and DELE with Fas receptor related DISC.** AKT/PKB: protein kinase B, CARD: caspase activation and recruitment domain, DAP3: death-associated protein 3, DD: death domains, DED: death effector domains, DELE: death ligand signal enhancer, DR: death receptor, DISC: death inducing signalling complex, FADD: Fas Associated Death, FLICE: FADD-like interleukin-1 beta-converting enzyme, FLIP: FLICE inhibitory protein.

A similar role in coordinating downstream effects of DRs is seen in the context of death-associated protein 3 (DAP3). DAP3 is a highly conserved GTP-binding protein of 4 kDa, encoded by the *DAP3* gene located in chromosome 1 q21 [24]. It is normally kept inactive as phosphoprotein by the action of protein kinase B (AKT/PKB). When activated, it co-localises with FADD and participates in the formation of the DISC [25]. Initially evidence suggested the DAP3 is involved in the actions of TNF-α, Fas-L, and TRAIL. However, more recent studies are suggestive that its association is strongest with the Fas receptor related DISC. In this context its actions are enabled by binding with death ligand signal enhancer (DELE), a relatively novel protein whose knockdown in HeLa cells was found to inhibit apoptosis due to TNF- α, Fas or TRAIL (Figure 1) [26].

In a similar fashion, DAP3 was found to mediate interactions between FADD and LKB1 interacting protein 1 (LIP1) in human osteosarcoma cells, which anchors LKB1 to the cytoplasm. This study characterised the expression of LKB1 to be critical for TRAIL-induced apoptosis mediated by DAP3 on the basis of knock-down studies in osteosarcoma cells [27]. LKB1 has been previously studied with regards to its role in Peutz-Jeghers syndrome. It is characterised as a serine-threonine kinase, with a predominantly tumour-suppressant role in contrast to the mTOR pathway [28,29].

Furthermore, DAP3 has been shown to be critical for the mediation of anoikis, which is to be discussed at greater length below [25].

### Intrinsic pathway

This pathway is influenced by a large array of non-receptor factors which may positively or negatively influence the activation of apoptosis. These factors include but are not limited to toxins, hormones, viral infections, physical stimuli and free radicals. The major strand of this pathway involves the release of mitochondrial contents due to changes in mitochondrial outer membrane permeabilisation (MOMP) [30].

### BCL2 family and control of mitochondrial outer membrane permeabilisation (MOMP)

MOMP is directly controlled by the BCL2 family of proteins. The BCL2 family of protein are defined by the presence of BCL2 homology motifs (BH1 to BH4). These domains were initially seen in BCL2, the archetypical member of this group of proteins. BCL2 was initially identified as an oncogene in B-cell lymphoma cells [30]. This was followed by the discovery of BAX, which was characterised as an anti-oncogenic protein [31]. Since then, around two dozen members of the BCL2 family have been identified, with variable tissue expression and differences in expression of BH domains [32].

Broadly speaking, BCL2 proteins can be classified as either pro-apoptotic or anti-apoptotic proteins. In turn the pro-apoptotic molecules are divided into multi-domain molecules, and BH3-only molecules. A precise model for the relationship between classes of BCL2 molecules is yet to be devised, with up to four enumerated by Shamus-din *et al.* The consensus seems to be that in normal conditions the multi-domain pro-apoptotic molecules (BAX, BAK, BOK) are sequestered in combination with anti-apoptotic molecules (BCL2-like, including BCL-xL, BCL-w and others). When this pathway is activated, BH3-only molecules (BID, PUMA, NOXA) bind with the BCL2-like molecules, allowing the multi-domain pro-apoptotic molecules to localise to the mitochondrial outer membrane. BAX/BAK oligomerise to form pores in the membrane, causing MOMP, which leads to disgorging of intra-mitochondrial contents [33].

The BH3 molecules have been identified as the control element of this process. Their expression and activation seems to be influenced by a variety of stimuli. p53 is the most significant up stream activator for the BH3 molecules [34,35].

The first wave of mitochondrial apoptogenic agents include cytochrome c, the second mitochondria-derived activator of caspase/direct IAP binding protein with low Pi (SMAC/DIABLO) and high temperature requirement A2/Omi (HTRA2/OMI).

### Cytochrome c and the apoptosome recruitment complex

Cytochrome c interacts with apoptotic protease activating factor 1 (APAF1) in the presence of ATP or dATP to form a multimeric complex referred to as an apoptosome. APAF1 has a DD termed the caspase activation and recruitment domain (CARD). [36]. CARD enables interaction with and activation of pro-caspase 9, leading to the production of activated caspase 9 [37,38].

### Inhibitors of apoptosis proteins (IAPs), SMAC/DIABLO and HTRA2/OMI

The activation of the caspase cascade has been characterised as an all or none outcome. However, the activation of caspases is normally prevented by the inhibitors of apoptosis proteins (IAPs). Upon invocation of the internal apoptotic pathway, factors sequestered in the mitochondria are released which include, SMAC/DIABLO [39]. SMAC/DIABLO inhibits the activity of IAPs, especially that of X-linked IAP (XIAP) by sequestering it [40]. Furthermore, Yu *et al.* suggest that SMAC/DIABLO sensitises cells to apoptosis induced by PUMA, thus providing a positive feedback for apoptosis [41]. This may be partially balanced by a recently described function of XIAP, in which it impairs the release of SMAC/DIABLO [42].

Similarly, MOMP also leads to the release of HTRA2/OMI, a serine protease which binds and cleaves various IAPs [43]. Additionally, a proteome-wide analysis suggests that HTRA2/OMI may also target cytoskeletal proteins [44].

### Mitochondrial DNA-ases

Further mitochondrial contents are then released which contribute to DNA breakdown. These include apoptosis inducing factor (AIF) and endonuclease G (EndoG).

AIF binds to DNA in a sequence-nonspecific manner, inducing chromatin condensation and initiating DNA breakdown [45,46].

EndoG has been demonstrated to form a complex with histone H2B and human DNA topoisomerase II alpha (TOPO2a) after translocation to the cytosol. Whist the details are as yet undetermined, this complex is believed to mediate the apoptosis-associated breakdown of DNA [47].

### Pro-survival role of DAP3 in mitochondrial function

Counter-intuitively, DAP3 has a pro-survival role in mitochondrial function. There are believed to be distinct intra-mitochondrial and cytosolic pools of DAP3, with the former resulting from the translocation of the latter through the mitochondrial membrane. The mitochondrial DAP3 forms an important portion of the 28 S subunit of the mitochondrial ribosome, thus essential for mitochondrial protein synthesis [48]. Furthermore, Kim *et al.* reported a deficiency of cytochrome c oxidase 1 in the mitochondria of *Dap3* −/− mouse embryos. As this is a protein encoded in the mitochondrial genome, it could be inferred that DAP3 has an integral role in intra-mitochondrial protein synthesis [49].

The duality seen in the intra-mitochondrial and cytosolic roles of DAP3 is not unlike other mitochondrial proteins, such as cytochrome c and IAP.

### PIDDsomes

p53 coordinates DNA repair and may induce apoptosis via various pathways. In addition to induction of BH3-only BCL2 proteins, p53 is known to induce the expression of the protein induced by p53 with a death domain (PIDD). PIDD forms a complex with the RIP associated ICH1/CED homologous protein with death domain (RAIDD). RAIDD is an adaptor protein which also possesses a CARD motif. This enables it to recruit pro-caspase 2. This complex is referred to as the PIDDsome, and is believed to mediate apoptosis in response to DNA damage. The active caspase 2 is formed by conformational changes in the pro-caspase induced by dimerization. Caspase 2 is an apical caspase, which activates further components of the caspase cascade [50,51].

### Common execution pathway: the caspase cascade

Intra- and extra-cellular signals converge on a common pathway which mediates the catabolic processes that constitute apoptosis. This pathway consists of a cascade of cysteinyl aspartic acid-protease, commonly referred to as caspases. Up to 14 have been enumerated in human and mammalian species [15]. They broadly fall into three groups:

### Apical (Initiators)

As detailed above in relation to the discussions regarding the relevant recruitment complexes, the apical caspases include caspases 2, 8, 9, and 10. These pro-enzymes have DDs, which coordinate the pro-caspases with DISC components with similar domains. This interaction induces dimerization and conformation change in the pro-caspases, leading to transformation to active caspase, as well as, auto-activation of the caspases [37,52].

With regards to caspase 8, Scaffidi *et al.* postulate that cells may be classified into two types regarding apoptosis. Type 1 have been characterised as cell which only require the action of caspase 8 to undergo apoptosis, whist type 2 cells which would also require MOMP to successfully undergo induced cell death [53].

When activated, they cleave downstream caspases, such as caspases 3 and 7. Also, they further stimulate upstream apoptosis processes. For instance, caspase 8 is known to cleave BH3 interacting domain death agonist (BID) to yield truncated BID (tBID). tBID increases MOMP, thus further committing the cell towards cell death [54]. Similarly, caspase 8 is known to cleave XIAP [55].

### Effectors (Executioners)

Caspases 3, 6 and 7 are activated by the apical caspases, and mediate the effects of the caspase cascade.

Caspase 3 is the most significant of the effector caspases, and can be activated by caspases 8, 9 and 10. It shares a lot of substrates with caspase 7. When activated, it mediates breakdown of DNA and of the cytoskeleton, degradation of XIAP, activation of caspase 6, as well as activation of the rest of the caspase cascade [56].

Caspase activated DNA-ase (CAD), also known as DNA fragmentation factor, 40 kDa, beta polypeptide (DFFB), normally exists in complex with inhibitor of CAD (ICAD). When activated, caspase 3 cleaves ICAD, leaving CAD unimpeded to breakdown DNA [57,58]. Upon activation, caspase 3 is believed to participate in further activation of the other components in the apoptosis pathway. It cleaves BID, thus further activating the permeabilisation of the mitochondrial outer membrane [56].

Caspase 3 also inhibits XIAP, thus further potentiating the action of caspase 9 [59].

Furthermore, caspase 3 cleaves gelsolin, which has a role in actin polymerisation and signal transduction [60].

Aminophospholipid translocase normally mediates an ATP-dependent process in which phosphotidyl serine (PS) is transferred from the outer layer of cell membrane to the inner layer. An accumulation of PS on the outer surface which occurs in the course of apoptosis, marks the cell for phagocytosis [61]. Studies in erythrocytes implicate caspases 3 and 8 in inactivation of the aminophospholipid translocase [62].

On the other hand, caspase 6 has a distinct substrate specificity *versus* caspases 3 and 7 which is currently being delineated. Of note, caspase 6 is being studied regarding it avidity for lamin proteins of the nuclear envelop [63].

### Inflammatory caspases

Caspase 1, 4, 5, and 11 have been found to have roles in immune response and innate immunity.

Specifically, they are activated by recruitment complexes composed of nucleotide-binding domain (NB) and leucine-rich repeat (LRR) containing receptors (NLR). Especially noteworthy of this group of proteins are NLRP1 and NLRP3, which form complexes with adaptor protein PYDCARD. These complexes are known as inflammosomes. These inflammosomes recruit caspases 1 and 5 respectively [64]. Inflammosomes have been implicated in immunity and more recently, metabolic syndrome [65].

Similarly, caspase 11 is also recruited by inflammosomes in the context of immune response to intracellular pathogens [66].

Caspase 4 has been implicated in NLR signalling and the activation of inflammosomes [67,68]. In addition, it also may be involved in mediation of endoplasmic reticulum stress related apoptosis [69-71].

### Perforin/Granzyme pathway

This is a relatively novel pathway utilised by immune cells in addition to Fas-Fas ligand interactions. In this case the T and NK cells insert pore molecules into the cell membrane of the targeted cell, which have been termed perforins. The perforins interact with intracellular secretory granules (SGs) which release granule associated enzymes (granzymes) A and B [72].

Granzyme A activates DNAase NM23-H1, which destroy nuclear material. This DNAase is normally bound to the SET complex, which is cleaved by granzyme A leading to the release and activation of the said DNAase [73].

Granzyme B activates pro-caspases 3 and 10 and cleaves ICAD. In addition, it activates the intrinsic pathway by inducing cytochrome c release and by cleaving BID protein, a member of the BCL2 family [74,75].

### Anoikis

Anoikis was initially described by Frisch *et al.* as apoptosis resulting from the detachment of epithelial cells from surrounding inter-cellular matrix and from surrounding cells. It is believed to be a cellular programme aimed preservation of normal tissue structure from disruption by inappropriate cell growth and migration. In the course of oncogenesis, neoplastic cells would inevitably have to overcome this cellular programme as part of the epidermal-mesenchymal transition [16].

**Figure 2:**
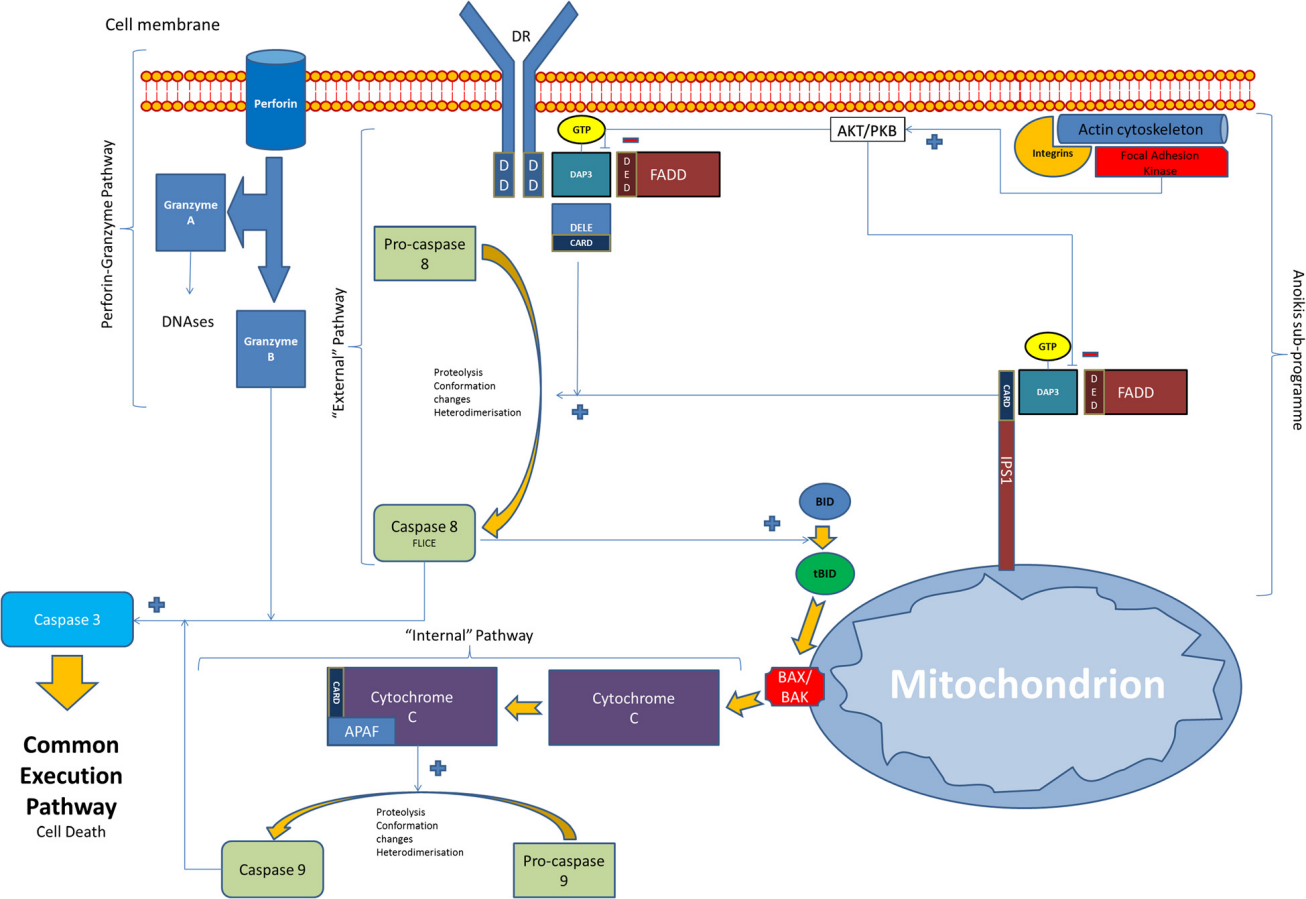
**General scheme illustrating the internal, external and perforin/granzyme pathways within apoptosis, including the sub-programmes specific to anoikis and DAP3.** AKT/PKB: protein kinase B, CARD: caspase activation and recruitment domain, DAP3: death-associated protein 3, DD: death domains, DED: death effector domains, DELE: death ligand signal enhancer, DR: death receptor, DISC: death inducing signalling complex, FADD: Fas Associated Death, FLICE: FADD-like interleukin-1 beta-converting enzyme, IPS: 1interferon-β promoter stimulator 1.

Our understanding of this cellular programme is currently evolving. Different cell types require specific integrin to extracellular matrix (ECM) interactions. Furthermore, the internal signalling of these interactions into the apoptosis pathways may vary between cell types, and are currently being delineated. These signals are believed to be largely channelled through the internal apoptosis pathway, with some involvement of the external pathway, culminating in the common execution pathway (Figure 2) [76].

### Role of the internal pathway in anoikis

The signalling from integrins requires mediation by focal adhesion kinase (FAK) which is believed to inhibit p53, which would induce apoptosis if the action of FAK or contact with ECM were lost. Ilic *et al.* have determined that the p53-induced apoptosis is susceptible to inhibition by BCL2 [77,78]. Studies by Chen *et al.* found that changes in cell morphology can also induce anoikis, and that the integrins, FAK and the actin cytoskeleton of the cell all play roles in anoikis as part of a focal adhesion complex [79].

Pro-survival signals, including those from anoikis, are believed to be passed by the extracellular-related kinase 1 and 2 (ERK1/2) and AKT/PKB are known to interact with BH3 BCL2 proteins, in particular BIM [80]. Similarly, the RAF pathway, the mitogen/extracellular kinase pathway (MEK) and MAP kinase pathway are also known to transmit such signals, as shown in studies in lung fibroblasts [81]. Puthalakath *et al.* have identified a role similar to that of BIM for BMF in the context of anoikis [82].

Idogawa *et al.* have characterised BAD as a specific BH3 molecule for anoikis. Whilst it did not initiate anokis, it did increase sensitivity to loss of AKT/PKB signalling [83].

Removal of such signals results in activation of the BH3 proteins, which sequester the anti-apoptotic BCL2 proteins, thus shift the balance towards BAX-BAK pore formation and initiation of MOMP, formation of the apoptosome, and activation of caspase 9 [84].

### Role of the external pathway in anoikis, including that of DAP3

A role for the external pathway in anoikis was proposed initially by Frisch *et al.*, and has been the subject of much study. The role of caspase 8 and Fas-DISCs in the propagation of the apoptosis cascade was identified earlier on [85]. In a study in colonic cells, Grossman *et al.* characterised the activation of caspase 8 to be a late event in anoikis, which was not likely to be the initiator of the cascade [86]. However, more recent studies in lymphocytes, oesophageal squamous cell and breast cancer cells characterise a FAK-caspase 8 axis central to anoikis signalling [87-89]. Studies in Jurkat cells have suggested that integrins, specifically β1 integrin, forms a complex with AKT/PKB, which inhibits pro-caspase 8 via PI3K. Loss of this inhibition is believed to be an initiator for anoikis [90].

The activation of caspase 8 in the context of anoikis has been associated with interactions with FADD of the Fas-related DISC. This interaction is inhibited by FLIP, which competes for FADD. This function of the Fas-related DISC appears to be ligand-independent [20]. DAP3 has been characterised as critical for anoikis. Upon the loss of the influence of AKT/PKB, it is dephosphorylated and interacts with FADD [25].

**Figure 3:**
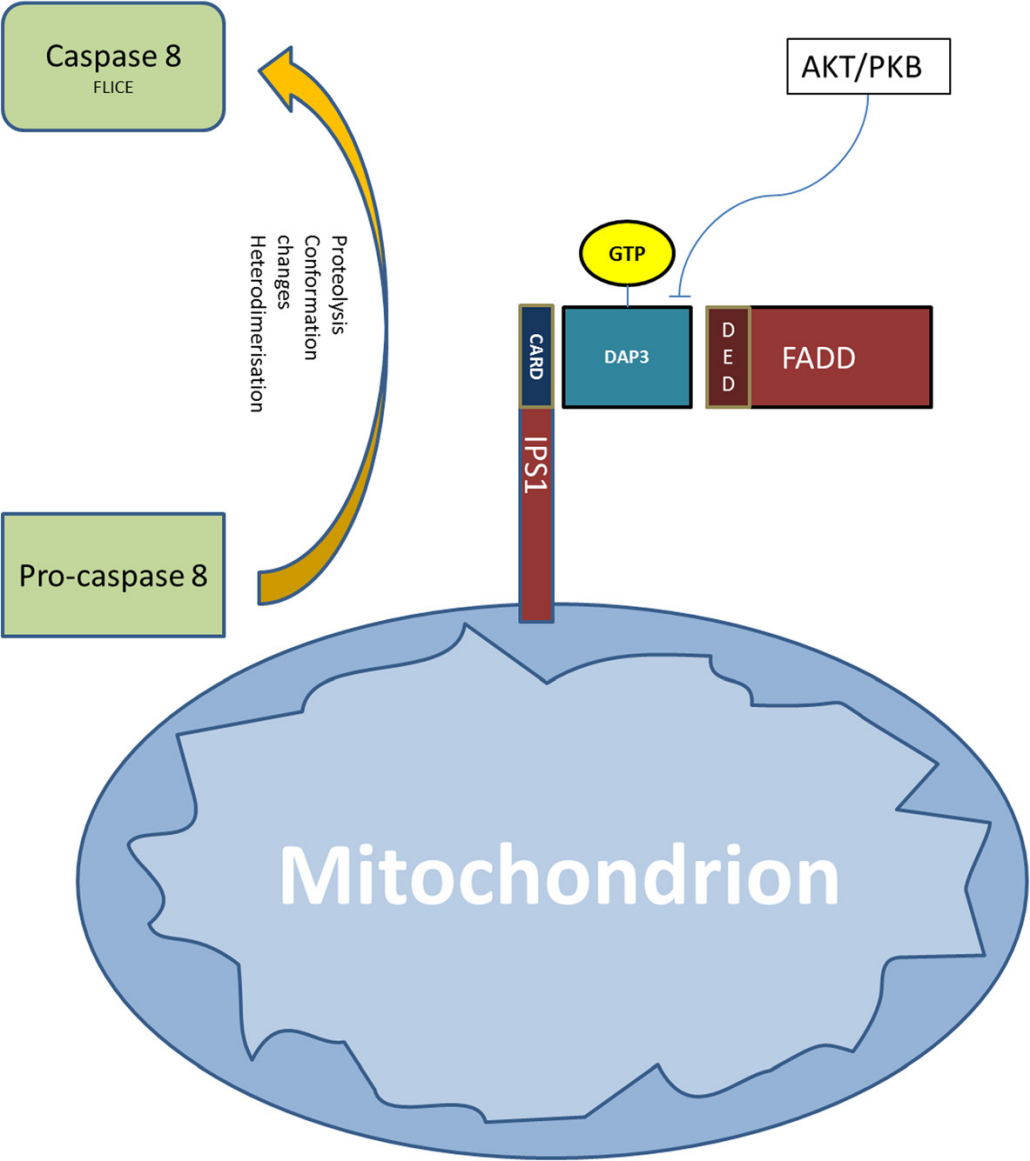
**Schematic representation of the relation of DAP3 and IPS1 on mitochondrial surface.** AKT/PKB: protein kinase B, CARD: caspase activation and recruitment domain, DAP3: death-associated protein 3, DD: death domains, DED: death effector domains, DELE: death ligand signal enhancer, DR: death receptor, DISC: death inducing signalling complex, FADD: Fas Associated Death, FLICE: FADD-like interleukin-1 beta-converting enzyme, FLIP: FLICE inhibitory protein, IPS: 1interferon-β promoter stimulator 1.

The FADD-DAP3 interaction requires the involvement of interferon-β promoter stimulator 1 (IPS1), which is a CARD bearing protein anchored on the mitochondrial outer membrane. It is a component of the innate immune response for viral infections, and communicates with inflammatory pathways. IPS1 localises the reaction to the mitochondrial membrane and recruits pro-caspase 8, thus triggering the caspase cascade (Figure 3) [91].

## Conclusion

### Death associated protein 3 and cancer

In conclusion, the current literature places DAP3 at the nexus of several highly significant and occasionally antagonistic pathways. However, there is scant literature with regards to its role in human neoplastic disease. It could be expected that DAP3 would have a tumour suppressant role predicated on its function as a pro-apoptotic molecule. However, DAP3’s countervailing pro-survival role in mitochondrial protein synthesis reduces such assumptions to the level of speculation.

Indeed, Mariani *et al.* reported increased expression levels of DAP3 in invasive glioblastoma tumour cells and in glioma cell lines with induced migratory phenotype [92]. These observations could be an instance of a neoplastic disease in which the mitochondrial maintenance prevails over the pro-apoptotic functions of the DAP3. Furthermore, this could be explained by the fact that P-loop mutant DAP3 is less effective in inducing apoptosis, and the COOH-terminal deleted protein (230 amino acids) acts in a dominant negative fashion, protecting cells from induced apoptosis [24].

On the other hand, our group has published a clinical study, in which we examined the mRNA expression of DAP3 in a breast cancer cohort of 127 patients, with a median follow-up of 10 years. Specifically we recorded a strong inverse correlation between mean copy number of DAP3 and tumour grade, Nottingham Prognostic Index, clinical stage, and clinical outcome. Furthermore, Kaplan-Meier analysis shown better survival in the high transcription group approaching significance [93].

Further investigations would be required to characterise the role of DAP3 in human breast cancer. We have presented our initial findings regarding studies into cell behaviour of knock-down sub-lines of MCF7 and MDA-MB-231 at the Scientific Meeting of British Association of Surgical Oncology in London, and have recently published our final results. We found that DAP3 knockdown sub-lines of MCF7 and MDA-MB‑231 had significantly increased adhesion and decreased growth when compared to the controls. Furthermore, invasion and migration were significantly increased in the MDA-MB-231^DAP3kd^ cells when compared to controls. The results of the adhesion, migration and invasion assays are supportive of the hypothesis of DAP3 as a pro-apoptotic antioncogenic protein, whilst the countervailing effect on growth may be due to pro-survival role of DAP3 in mitochondrial protein synthesis [94].

In view of the locus of DAP3 within several cellular pathways of importance in oncogenesis, we believe that DAP3 would be a very fruitful avenue for further research. Such research would hopefully engender a better understanding of underlying pathways, enabling more discrete therapeutic targeting.

## Abbreviations

ACD: Accidental cell death
AIF: Apoptosis inducing factor
AKT/PKB: Protein kinase B
APAF1: Apoptotic protease activating factor 1
BAX: BCL2 associated X protein
BCL2: B-cell lymphoma 2 protein
BID: BH3 interacting domain death agonist
CAD: Caspase activated DNA-ase
CARD: Caspase activation and recruitment domain
caspases: Cysteinyl aspartic acid-proteases
DAP3: Death-associated protein 3
DD: Death domain
DED: Death effector domains
DELE: Death ligand signal enhancer
DISC: Death inducing signalling complex
DR: Death receptors
ECM: Extra cellular matrix
EndoG: Endonuclease G
ERK1/2: Extracellular-related kinase 1 and 2
FADD: FAS Associated Death Domain
FAK: Focal adhesion kinase
FLICE: FADD-like interleukin-1 beta-converting enzyme
FLIP: FLICE-like inhibitory protein
granzymes: Granule associated enzymes
HTRA2/OMI: High temperature requirement A2/Omi
IAPs: Inhibitors of apoptosis proteins
ICAD: Inhibitor of CAD
LIP1: LKB1 interacting protein 1
IPS1: Interferon-β promoter stimulator 1
MEK: Mitogen/extracellular kinase pathway
MOMP: Mitochondrial outer membrane permeabilisation
NFKB1: Nuclear factor of kappa light polypeptide gene enhancer in B-cells 1
NLR: Nucleotide-binding domain (NB) and leucine-rich repeat (LRR) containing receptors
PCD: Programmed cell death
PIDD: Protein induced by p53 with a Death Domain
PS: Phosphotidyl serine
RAIDD: RIP associated ICH1/CED homologous protein with death domain
SMAC/DIABLO: Second mitochondria-derived activator of caspase/direct IAP binding protein with low Pi
tBID: Truncated BH3 interacting domain death agonist
TNF: Tumour necrosis factor
TNFR: Tumour necrosis factor receptor
TOPO2a: Topoisomerase II alpha
TRAIL: TNF related apoptosis inducing ligand
TRADD: TNFRSF1A-associated via death domain
TRAF2: TNF receptor-associated factor 2
XIAP: X-linked IAP
